# Supplementary material for: Transcriptomic Insights into Mechanisms of Early Seed Maturation in the Garden Pea (Pisum sativum L.)
Source: Cells. 2020 Mar 23;9(3):779. doi: 10.3390/cells9030779 (PMC7140803; doi:10.3390/cells9030779)
Supplement: Supplementary file 1 [file cells-09-00779-s001.zip › Supplementary materials/FileS4.zip!/FileS3/TFs - Results/Supporting Maps&Profiles/Supporting Maps.pdf]

Population Map

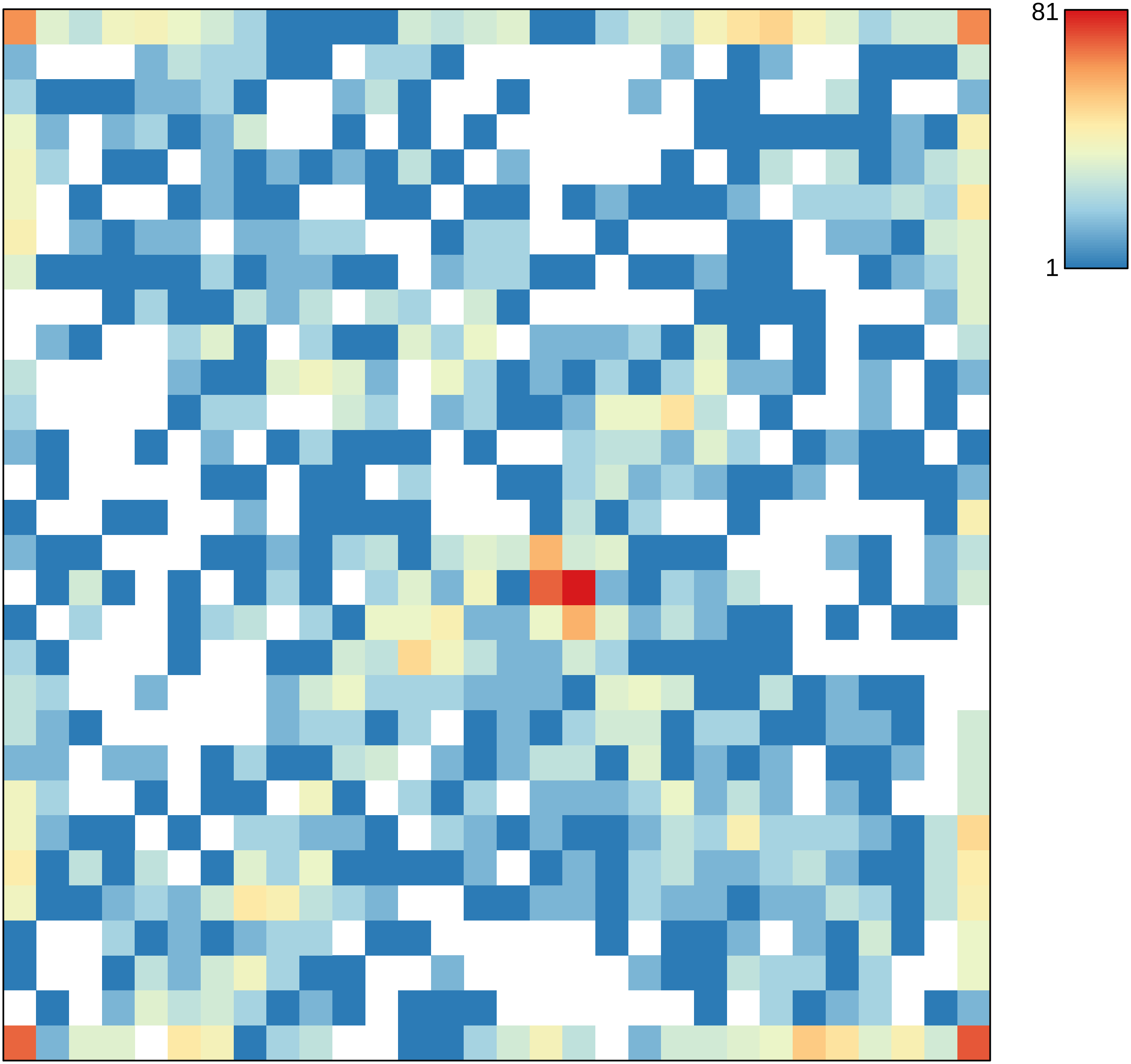

log (# genes in metagene)

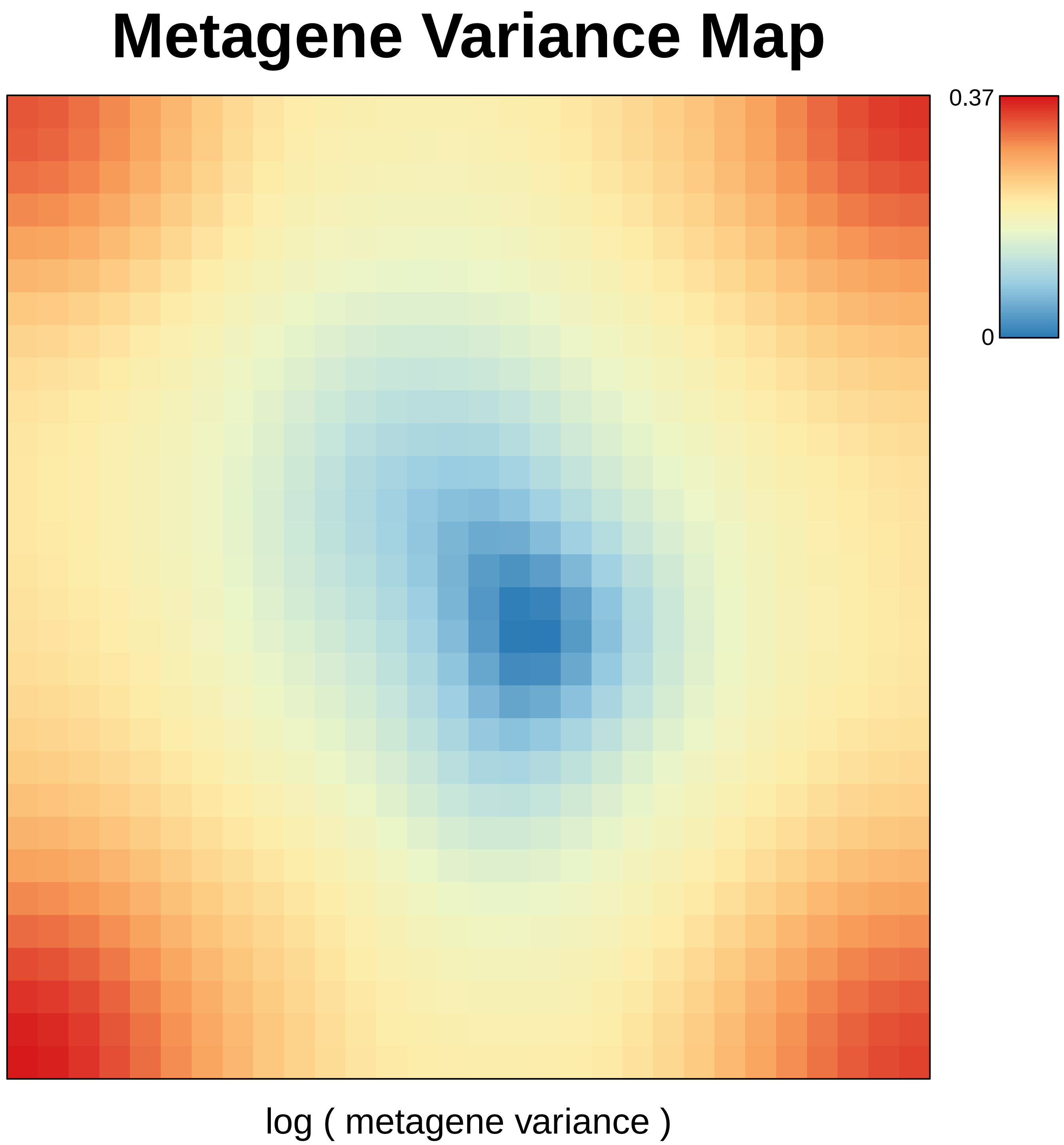
Metagene Variance Map
0.37
0
log (metagene variance)

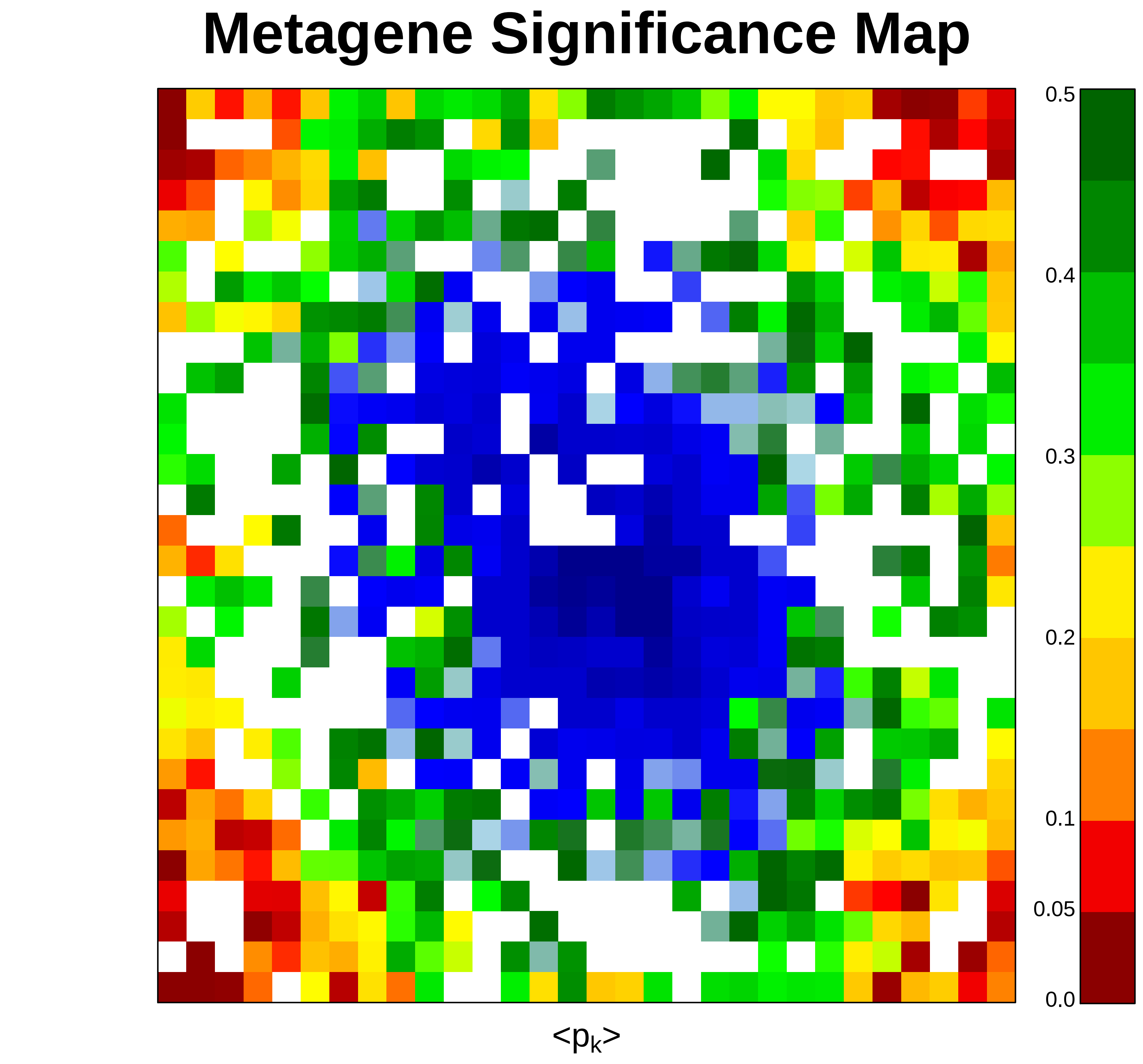

Metagene Significance Map
0.5
0.4
0.3
0.2
0.1
0.05
0.0
<p_k>

Metagene Significance Map

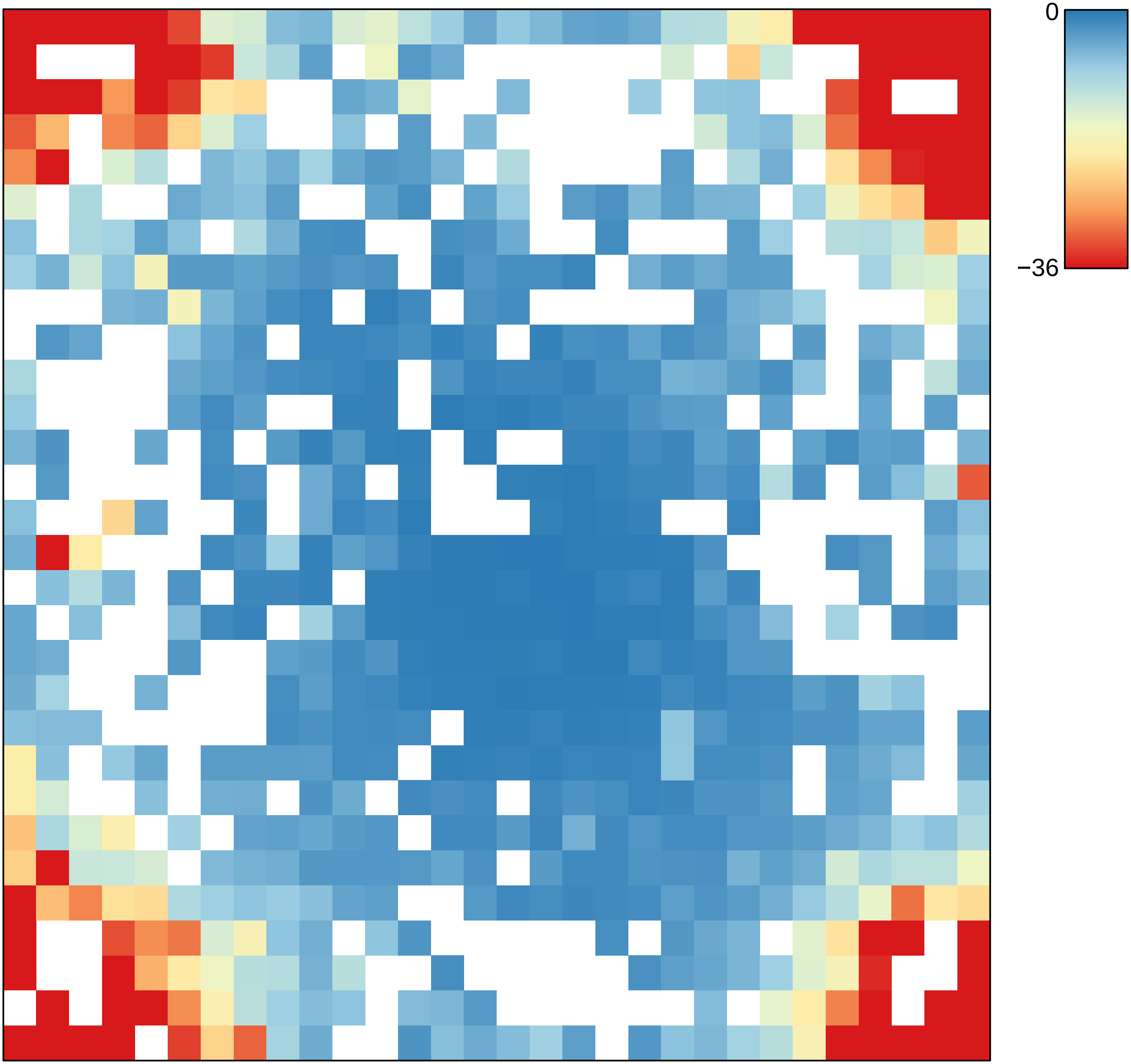

$\min_m(\log p_{k,m})$

Standard Metagene Entropy Map

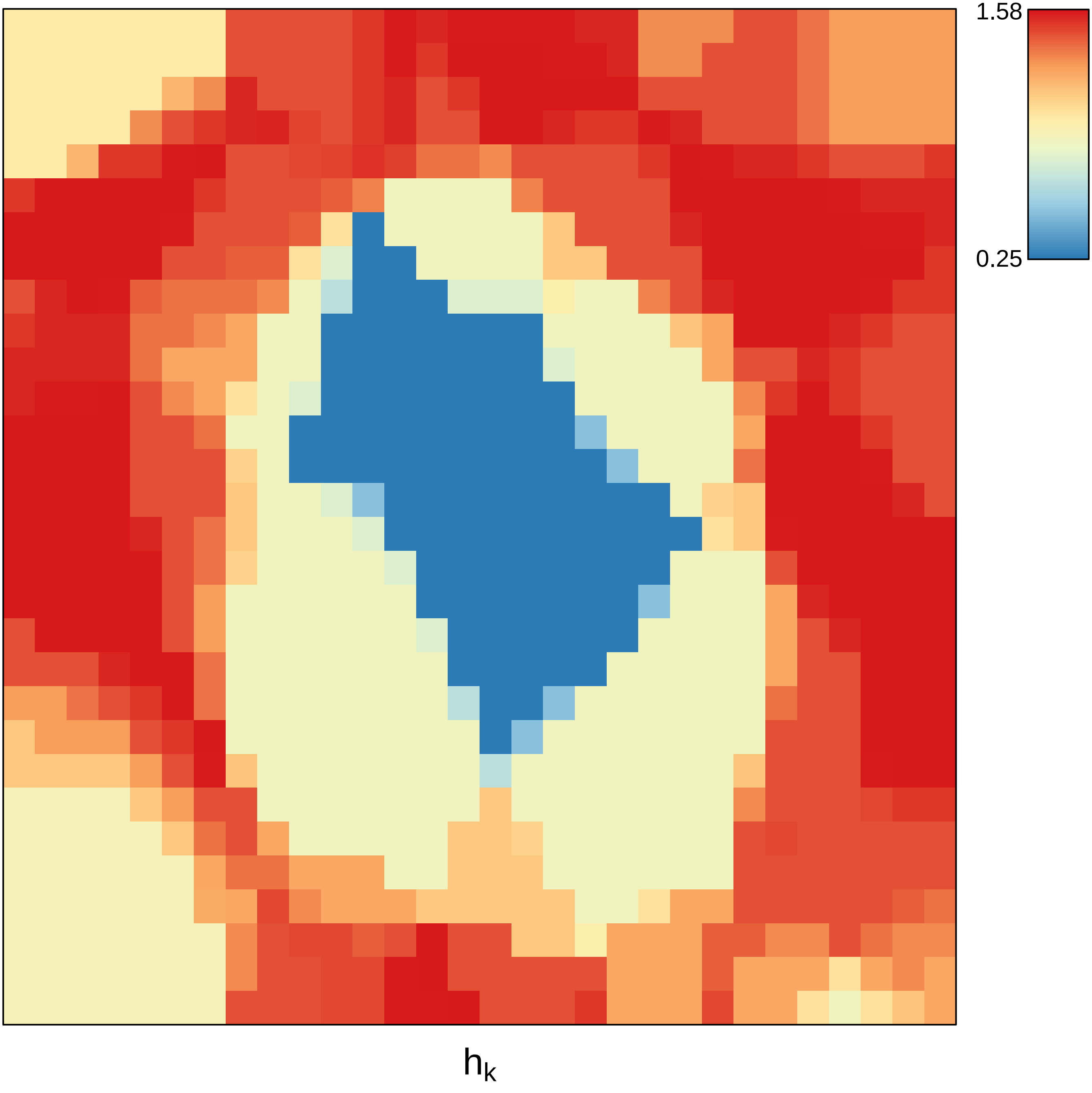

Gene−Metagene Covariance Map

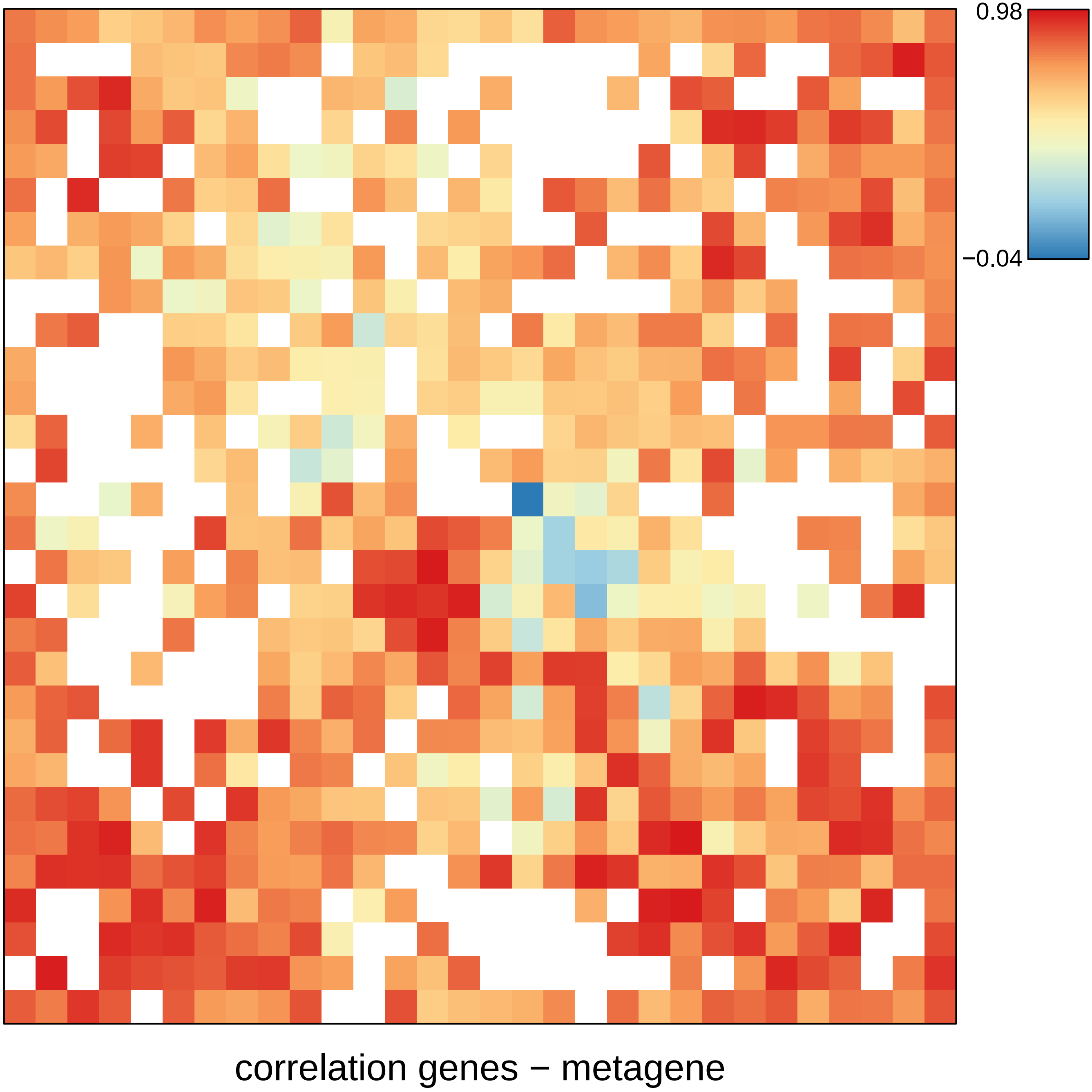

Deviation Map

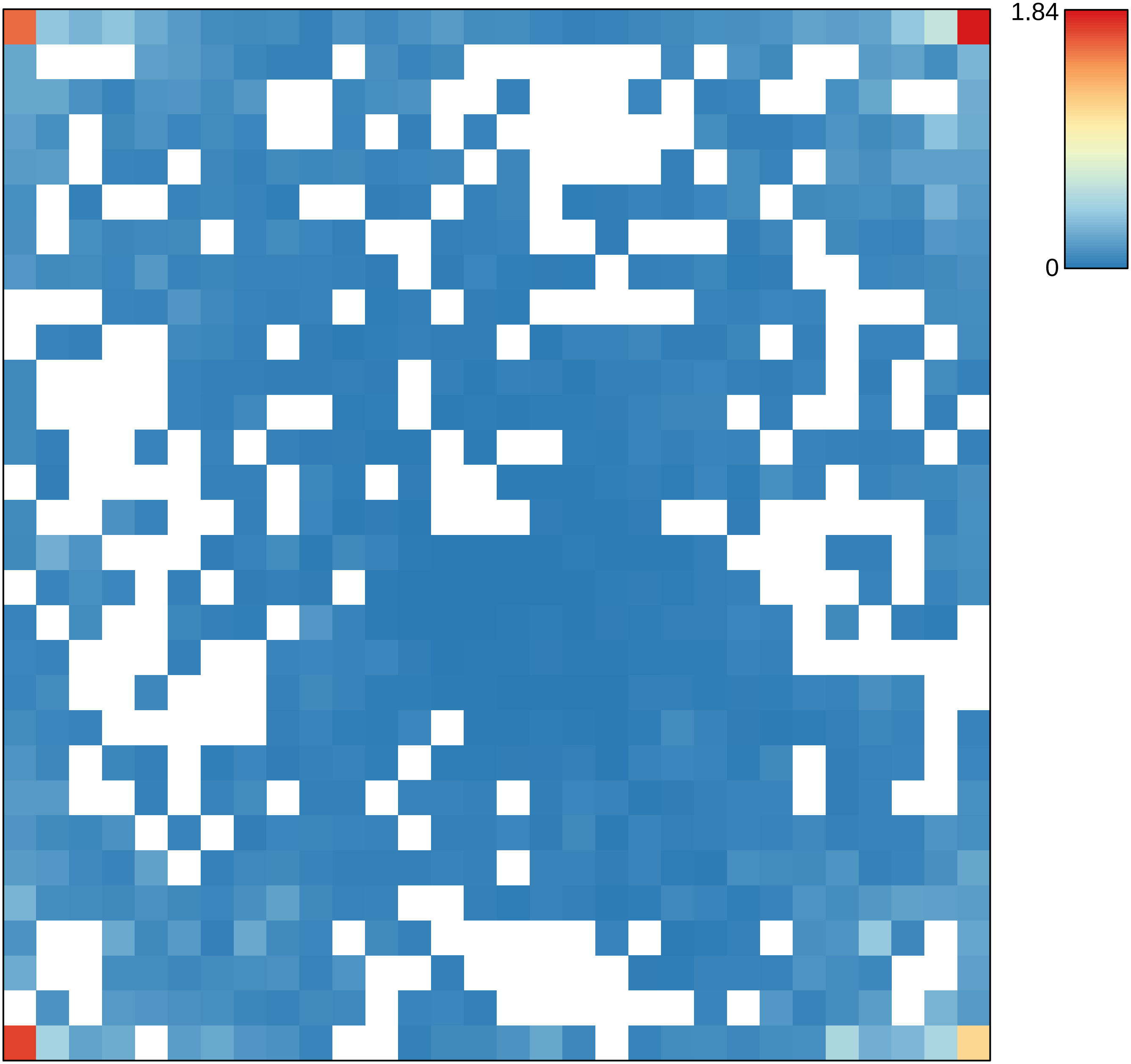

deviation genes − metagene

Distance Map

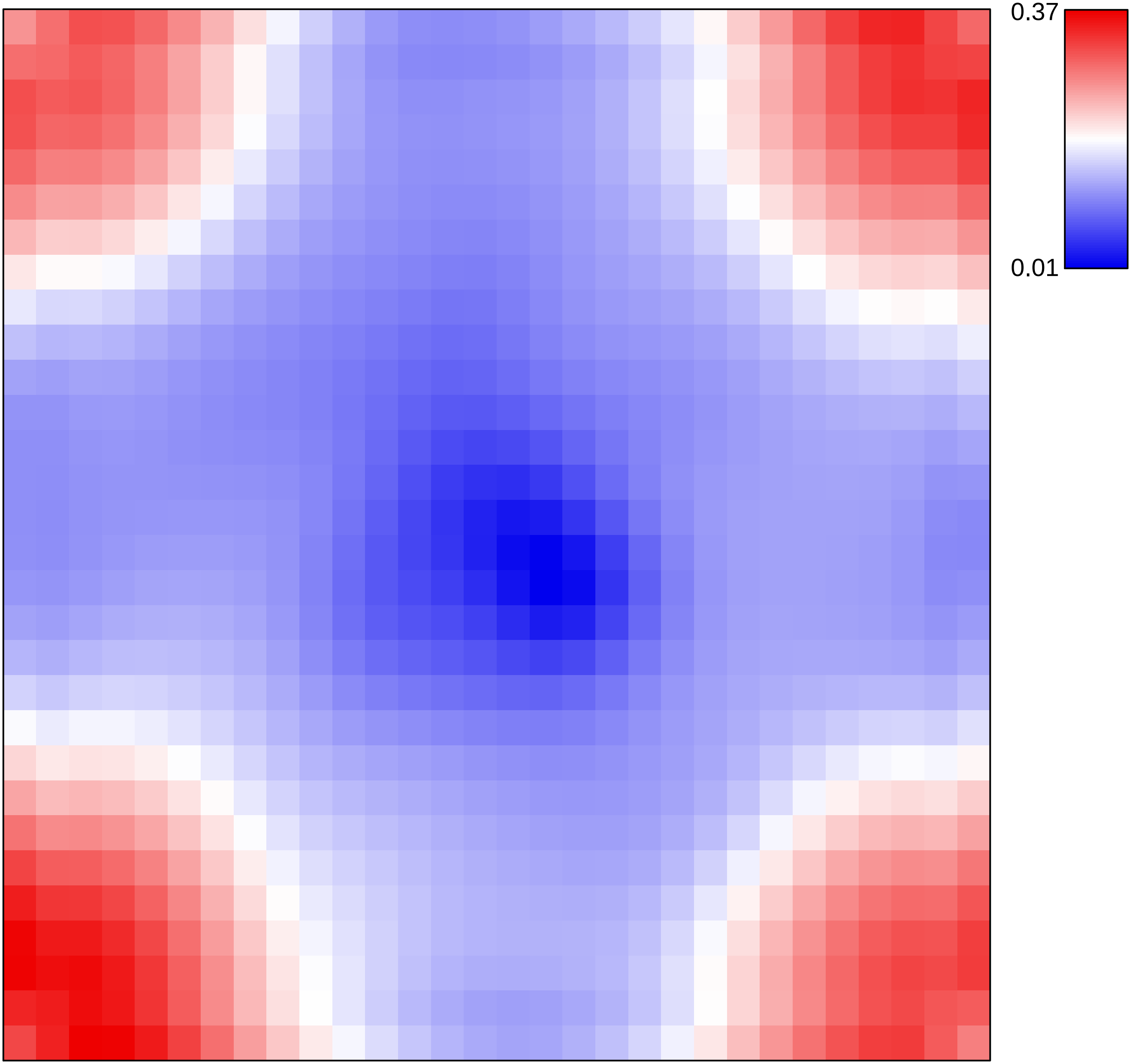

deviation of adjacent metagenes
